# Genotypic differences in intruder-evoked immediate early gene activation in male, but not female, vasopressin 1b receptor knockout mice

**DOI:** 10.1186/s12868-016-0310-7

**Published:** 2016-11-24

**Authors:** Shannah K. Witchey, Erica L. Stevenson, Heather K. Caldwell

**Affiliations:** 1 Laboratory of Neuroendocrinology and Behavior, Department of Biological Sciences, Kent State University, 256 Cunningham Hall, Kent, OH 44242 USA; 2 Laboratory of Neuroendocrinology and Behavior, School of Biomedical Sciences, Kent State University, Kent, OH USA

**Keywords:** Intermale aggression, Resident intruder test, Maternal aggression, c-FOS, EGR-1

## Abstract

**Background:**

The neuropeptide arginine vasopressin (Avp) modulates social behaviors via its two centrally expressed receptors, the Avp 1a receptor and the Avp 1b receptor (Avpr1b). Recent work suggests that, at least in mice, Avp signaling through Avpr1b within the CA2 region of the hippocampus is critical for normal aggressive behaviors and social recognition memory. However, this brain area is just one part of a larger neural circuit that is likely to be impacted in Avpr1b knockout (−/−) mice. To identify other brain areas that are affected by altered Avpr1b signaling, genotypic differences in immediate early gene activation, i.e. c-FOS and early growth response factor 1 (EGR-1), were quantified using immunocytochemistry following a single exposure to an intruder.

**Results:**

In females, no genotypic differences in intruder-evoked c-FOS or EGR-1 immunoreactivity were observed in any of the brain areas measured. In males, while there were no intruder-evoked genotypic differences in c-FOS immunoreactivity, genotypic differences were observed in EGR-1 immunoreactivity within the ventral bed nucleus of the stria terminalis and the anterior hypothalamus; with Avpr1b −/− males having less EGR-1 immunoreactivity in these regions than controls.

**Conclusions:**

These data are the first to identify specific brain areas that may be a part of a neural circuit that includes Avpr1b-expressing cells in the CA2 region of the hippocampus. It is thought that this circuit, when working properly, plays a role in how an animal evaluates its social context.

**Electronic supplementary material:**

The online version of this article (doi:10.1186/s12868-016-0310-7) contains supplementary material, which is available to authorized users.

## Background

The neuropeptide vasopressin (Avp) and its homologues have been implicated in the neural regulation of social behaviors in many species [1–8]. In mammals, Avp is known to modulate aggression, affiliation, and social recognition memory [4, 9, 10]. These effects are mediated by two centrally expressed Avp receptors; the Avp 1a receptor (Avpr1a) and the Avp 1b receptor (Avpr1b). While there has been a lot of focus on the behavioral effects mediated by the Avpr1a, there is a substantial amount of evidence indicating that the Avpr1b also plays a critical role in the modulation of social behaviors [2, 9, 11–13].

Work in Avpr1b knockout (−/−) mice has revealed that the Avpr1b is essential for normal displays of social recognition memory and aggressive behaviors directed towards a conspecific [2, 12–17]. Specifically, male Avpr1b −/− mice are unable to discriminate between a littermate versus a novel animal [18] or a novel female versus a familiar female [17]. In an olfactory-based preference test, male Avpr1b −/− mice spend equal amounts of time with urine soiled bedding as they do with clean bedding, thus failing to display a preference; though it is known that they can distinguish between male and female odors [16]. Female Avpr1b −/− mice have an abnormal Bruce Effect [19], which is the pheromonally-based pregnancy block observed when newly pregnant female mice are exposed to an unfamiliar male (or his odor). Avpr1b −/− females fail to terminate their pregnancies in the presence of an unfamiliar male, which suggests that they are not able to ‘remember’ the male with which they previously mated [14]. Avpr1b −/− males also have significant reductions in offensive and defensive aggressive behaviors, but have normal predatory aggression, and females have reductions in maternal aggression [15, 17, 20, 21]. While these behavioral effects are interesting, identification of where in the brain Avpr1b is mediating these effects as well as how this receptor fits in with a larger neural circuit is equally important.

In mice, rats, and humans central Avpr1bs are discretely localized, with prominence in the CA2 region of the hippocampus [22]. This region has been identified as being important to normal social behavior, as lesions to the CA2 region result in impairments in social recognition memory [23], and larger lesions of the hippocampus, that include the CA2 region, not only impair social recognition memory but also reduce aggression [24–26]. Furthermore, the genetic “silencing” of CA2 pyramidal cells using a Cre-dependent adeno-associated virus in a transgenic mouse line (Amigo2-Cre) also disrupts social recognition memory [27]. While these converging lines of evidence point to an important role for the CA2 region of the hippocampus in the modulation of social behavior, they do not make the functional link between the Avpr1b, CA2, and behavior. That link comes from recent studies from the laboratory of W. Scott Young, who found that reinstatement of the expression of the Avpr1b in the dorsal CA2 region in Avpr1b −/− mice results in increases in aggressive behavior [13] and the enhanced memory following the stimulation of Avpergic projections from the paraventricular nucleus of the hypothalamus (PVN) to CA2 is dampened by injection of an Avpr1b antagonist into the CA2 region [12]. Due to the restricted expression of the Avpr1b and the aforementioned behavioral data, we and others have hypothesized that Avpr1b expression in the CA2 region of the hippocampus may form a link between olfactory cues and social interactions [2, 22]. This would in turn aid in the formation and/or recall of memories related to social encounters, in particular those that are accessory olfactory based. These memories are likely important in helping an animal figure out their specific social context, which ultimately will affect several important behaviors, including social recognition memory and aggression.

Unfortunately, much of the neural circuitry involved in Avp’s modulation of aggression via the Avpr1b has remained elusive, but Avpr1b −/− mice are well suited for asking and answering this type of question. To determine what brain areas are differentially activated following exposure to a perceived threat we used Avpr1b −/− mice to quantify any intruder-evoked genotypic differences in immediate early gene (IEG) activation. As previously mentioned, Avpr1b −/− mice display little to no aggression in tests of maternal and intermale aggression [15, 17, 20, 21]. It is for this reason that we focused on what IEG activation occurred in these mice when they were simply exposed to an intruder. IEGs are activated as part of the first response to stimuli and thus serve as important indicators of neurophysiological activation [28]. However, IEGs are not expressed the same in every neuron or in the same context. For example, there are regional differences in c-FOS and early growth response factor 1 (EGR-1) expression following maternal aggression [29, 30]. Thus, in an attempt to account for potential differences in IEG expression, both c-FOS and EGR-1 protein expression were quantified. We *hypothesized* that there would be genotypic differences in intruder-evoked IEG expression in the neural circuitry known to be involved in maternal and intermale aggression.

## Methods

### Animals and housing

Adult male and female Avpr1b +/+ and Avpr1b −/− mice, generated from heterozygous breeding pairs in the Kent State University vivarium, were kept on a 12:12 light: dark cycle, with food and water provided ad libitum. At the time of weaning (18–21 days post-partum), tails were clipped to extract DNA and PCR was performed in order to determine the genotypes [see, 15, 20, 21]. All subjects were 2–6 months of age at time of testing and all experiments were conducted in accordance with the protocol approved by the Kent State University Institutional Animal Care and Use Committee.

### Aggression testing

Adult female Avpr1b +/+ (n = 5) and Avpr1b −/− (n = 6) mice were exposed to male intruders in a maternal aggression test. Initially, female experimental mice were housed in single-sex groups (up to four per cage) for 2 weeks to synchronize estrous cycles. Three days prior to mating, male bedding was added to the females’ cages to induce the Whitten effect; where male odors induce estrus and synchronize estrous cycles among females [31, 32]. An experienced adult C57BL/6J breeder male from our animal colony was then placed in the cage for 1 week and females were checked daily for sperm plugs and parturition was estimated. One week prior to parturition, the females were single-housed and no cage changes performed. Following parturition, on postnatal day (PND) 2 l were culled to four pups. The details of the maternal aggression testing, which was performed on PND4 can be found below; this time point was selected because of previous work by us and by others [30, 33].

Similar to females, male Avpr1b +/+ (n = 12) and Avpr1b −/− (n = 10) mice were single housed for at least 2 weeks and with no cage change for 1 week prior to resident-intruder testing. Following isolation, the procedure for both tests was the same. Group-housed male Balb/c mice, a strain we have previously used as stimulus animals [21, 34], between the ages of 2 and 5 months were purchased from Jackson Laboratories (Bar Harbor, ME) and used as intruder animals. In order to acclimate both intruder and experimental animals to the testing space, all animals were moved to the behavioral testing room at least 1 h prior to testing and left undisturbed. Testing began approximately 1 h after lights out under dim red light illumination. Thus, testing was performed during the dark phase of the light: dark cycle. All sessions were recorded using an infrared camera. At the initiation of testing, an intruder was placed in the home cage of either a resident dam and its offspring (maternal aggression test) or the resident male (resident intruder test). Each experimental animal was exposed only once to an intruder and all experimental animals spent the same amount of time interacting with the stimulus animals—5 min (300 s). At the end of the 300 s the intruder was removed and returned to its home cage. Sixty minutes after the conclusion of the testing session, to allow time for the transcription and translation of c-FOS and EGR-1, all experimental animals were euthanized via cervical dislocation and decapitated. The brains of the experimental animals were immediately removed and immersion-fixed in 4% paraformaldehyde prior to being sectioned for immunohistochemistry (described below). All sessions were videotaped and watched by an experimenter blind to the experimental animals’ genotypes. If any animals had attacked they would have been removed from the experiment, as the motor components of those interactions could create an experimental confound. Fortunately, in our experiments, no animals attacked, possibly due to their being tested only once. Thus, we are confident that any genotypic-dependent changes in IEG expression can be attributed to the detection and/or processing of the social cues.

### Immunohistochemistry for c-FOS and EGR-1

Brains were fixed in 4% paraformaldehyde, cut at 50 μm using a Vibratome 1000 Plus (Leica Microsystems, Buffalo Grove, IL), separated into four sets of free-floating sections, and stored at −20 °C in cryoprotectant [500 ml 0.1 M potassium phosphate buffer, 300 g sucrose (37.5%), 10 g polyvinyl pyrolidione (0.0125%) and 300 ml ethylene glycol (37.5%)] prior to immunohistochemical staining. At time of staining, two serial series of tissue were washed in 1× phosphate buffered saline (PBS) six times for 10 min each, then incubated in 1.5% hydrogen peroxide (H_2_O_2_) for 5 min, and washed again in 1×PBS two times for 5 min each. Using Power Block™ Universal Blocking Reagent (BioGenex, San Ramon, CA), the tissue was blocked for 30 min. One of the two series was then incubated overnight in rabbit anti-c-FOS primary antibody (Santa Cruz Biochemicals, Santa Cruz, CA, USA, sc-52) at a dilution of 1:5000 in antisera diluent (1XPBS + 1% normal goat serum + 0.3% Triton X-100) at 4 °C [34]. The second set of tissue was incubated for 2 days in rabbit anti-EGR-1 primary antibody (Santa Cruz Biochemicals, Santa Cruz, CA, USA, sc-189) at a dilution of 1:10,000 in antisera diluent (same as described above) at 4 °C. The sections were then washed in 1XPBS three times for 5 min each to remove excess primary antibody. The tissue was then incubated for 1 h in biotinylated goat anti-rabbit secondary antibody (Vector Laboratories, Burlingame, CA) at a dilution of 1:500 in antisera diluent. Following incubation, the sections were washed in 1XPBS three times for 5 min each and exposed to an avidin–biotin complex for 1 h (Vector Laboratories, Burlingame, CA). The tissue was then washed in 1XPBS three times for 5 min each and the antibodies were visualized using diaminobenzidine (DAB). Sections were then washed in 1XPBS twice for 5 min to inactivate the DAB, mounted onto gel-subbed slides, dried overnight, counterstained with methyl green, and coverslipped.

### Quantification of IEG immunoreactivity

c-FOS-immunoreactive (ir) and EGR-1-ir cells were counted at 100× magnification by a single observer blind to testing groups. iVision software (BioVision Technologies, Exton, PA) was used to capture the images and immunoreactive cells were manually counted within each neuroanatomical area. Three sections per area were quantified, with sections being 100 μm apart. Counts were made bilaterally using set box sizes for each area (box sizes from [35]) and the counts averaged within each brain region (if there was poor staining on any sections, a minimum of three counts were required to generate an average that was included in the statistical analyses). The same areas were counted for both males and females. The areas quantified included the dorsal and ventral aspects of the lateral septum, the LSD (0.99 × 1.02 mm) and the LSV (0.52 × 0.46 mm), respectively, the dorsal bed nucleus of the stria terminalis (BNSTD) (0.82 × 1.18 mm) and the ventral BNST (BNSTV) (0.82 × 0.59 mm), the medial preoptic area (MPOA) (0.63 × 1.18 mm), the PVN (0.48 × 0.82 mm), the medial amygdala (MeA) (1.35 × 1.18 mm), the anterior hypothalamic area (AHA) (0.82 × 0.82 mm), and the CA2 region of the hippocampus (0.41 × 0.41 mm). These brains regions were selected because of their known role in aggressive behavior [5, 30, 36, 37] and were identified based on the Paxinos and Franklin mouse brain atlas [38]. All data were normalized by conversion to number of counts per mm^2^ as this adjusts for the various sizes of the boxes; it was these data that were statistically compared (see below). The raw data for all experiments can be found here (Additional file 1).

### Statistical analysis

As none of our animals attacked, all data from the maternal aggression test (Avpr1b +/+ n = 5 Avpr1b −/− = 6 for c-FOS and Avpr1b +/+ n = 5 Avpr1b −/− n = 5 for EGR-1 staining) and the resident-intruder test (Avpr1b +/+ n = 12 Avpr1b −/− = 10) were analyzed. One female was excluded in the EGR-1 staining due to experimenter error resulting in damage to the sections.

For each IEG examined for a particular brain area, comparisons were made within each sex between the genotypes using a one-way analysis of variance (ANOVA) (SPSS 22.0 for Mac, IBM, Armonk, NY). A result was considered statistically significant if p < 0.05.

## Results

### c-FOS-immunoreactivity

In both female and males there were no statistically significant genotypic differences in c-FOS-immunoreactivity/mm^2^ observed in any of the brain regions quantified. Means ± SEM for each group can be found in Table 1A, B.

**Table 1:** Mean number of c-FOS immunopositive cells/mm^2^ with standard errors for female (A) Avpr1b +/+ (n = 5) and Avpr1b −⁄− (n = 6) and male (B) Avpr1b +/+ (n = 12) and Avpr1b −⁄− (n = 10) mice exposed to an intruder male

Brain region	Avpr1b +/+	Avpr1b −⁄−
(A) Females		
LSD	79.4 ± 3.8	94.7 ± 22.7
LSV	249.3 ± 15.2	254.5.2 ± 20.4
BNSTD	128.8 ± 12.9	114.2 ± 9.6
BNSTV	144.3 ± 19.4	130.9 ± 11.9
MPOA	226.1 ± 34.6	205.8 ± 24.4
PVN	317.1 ± 59.4	298.3 ± 16.1
MeA	160.6 ± 26.5	154.9 ± 16.2
CA2	139.5 ± 8.4	151.3 ± 19.3
AHA	68.3 ± 16.4	66.7 ± 11.3
(B) Males		
LSD	76.1 ± 9.0	85.8 ± 11.8
LSV	243.7 ± 17.8	222.9 ± 20.4
BNSTD	96.0 ± 4.1	82.3 ± 7.6
BNSTV	133.9 ± 9.7	107.0 ± 12.7
MPOA	102.0 ± 8.5	103.7 ± 11.4
PVN	155.4 ± 16.5	167.2 ± 16.8
MeA	155.5 ± 15.0	158.2 ± 12.2
CA2	158.2 ± 17.9	134.6 ± 11.1
AHA	42.6 ± 5.0	45.0 ± 3.0

*LSD* dorsal lateral septum, *LSV* ventral lateral septum, *BNSTD* dorsal bed nucleus of the stria terminalis, *BNSTV* ventral bed nucleus of the stria terminalis, *MPOA* medial preoptic area, *PVN* paraventricular nucleus, *MeA* medial amygdala, *CA2* CA2 region of the hippocampus, *AHA* anterior hypothalamic area

### EGR-1-immunoreactivity

**Fig. 1:**
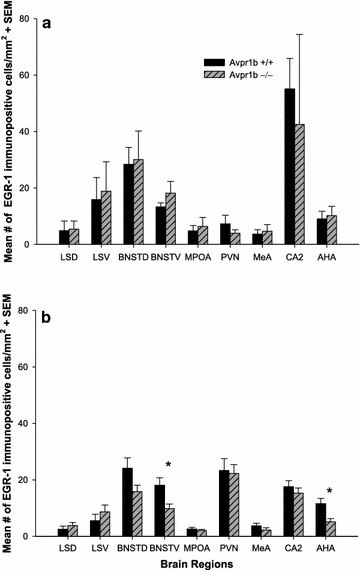
Mean number of EGR-1 immunopositive cells/mm^2^ in **a** female vasopressin 1b receptor wild type (Avpr1b +/+) and knockout (Avpr1b −/−) (n = 5 both groups) mice following a maternal aggression test and **b** male Avpr1b +/+ (n = 12) and −/− (n = 10) mice following a resident intruder test. In females, there were no genotypic differences in any of the brain areas measured. In males, there were genotypic differences in the ventral bed nucleus of the stria terminalis (BNSTV) (F_(1,20)_ = 6.89, p = 0.016) and anterior hypothalamic area (AHA) (F_(1,18)_ = 8.46, p = 0.009); with Avpr1b −/− males having fewer EGR-1 immunopositive cells/mm^2^ than wild type controls. *LSD* dorsal lateral septum, *LSV* ventral lateral septum, *BNSTD* dorsal bed nucleus of the stria terminalis, *MPOA* medial preoptic area, *PVN* paraventricular nucleus, *MeA* medial amygdala, *CA2* CA2 region of the hippocampus. *Asterisks* indicates a *p* value of less than 0.05

**Fig. 2:**
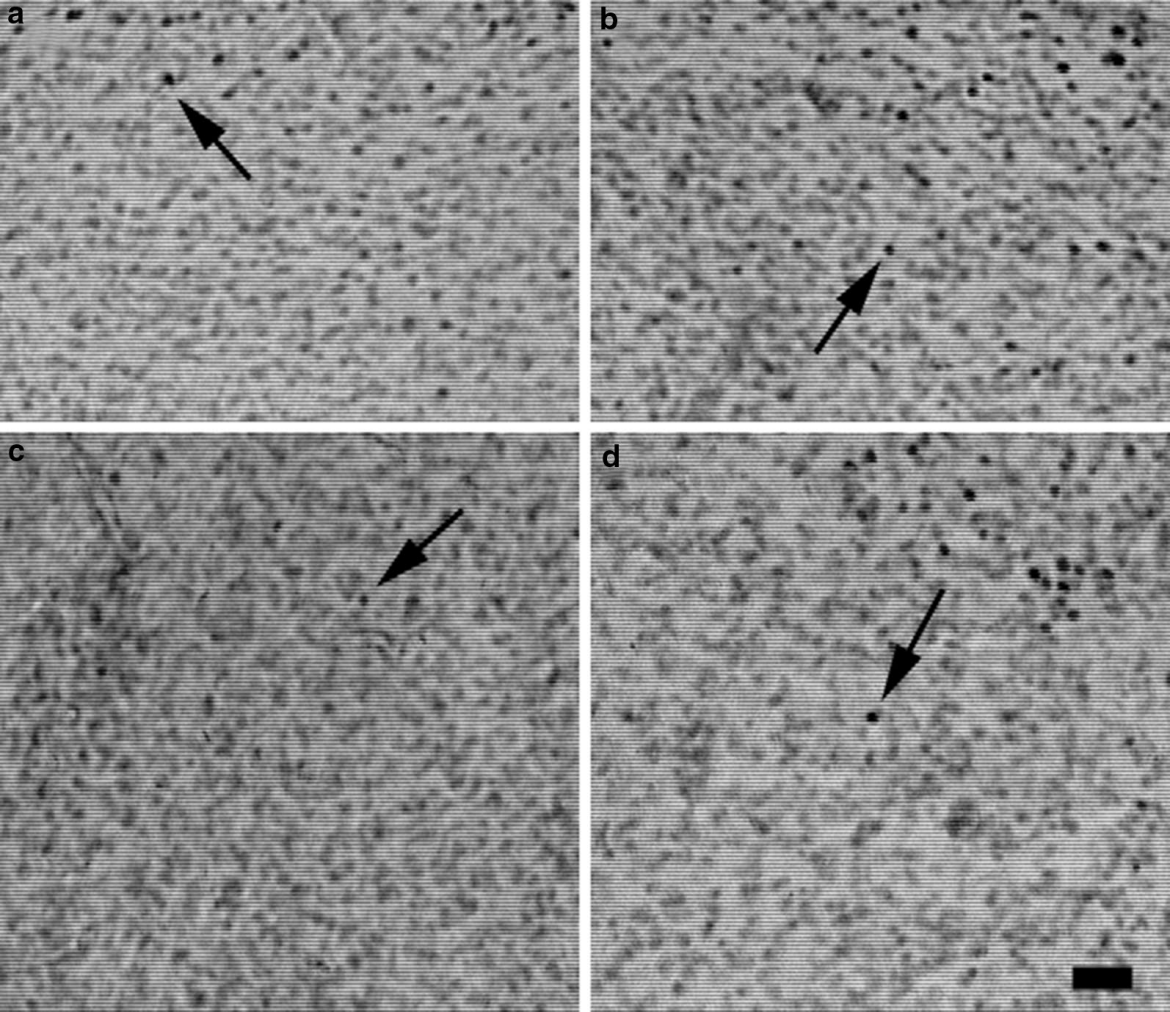
Intruder-evoked EGR-1 expression in vasopressin 1b knockout (Avpr1b −⁄−) and wild type (Avpr1b +⁄+) male mice. **a**, **b** Representative photomicrographs of the ventral bed nucleus of the stria terminalis (BNSTV) in Avpr1b −⁄− (**a**) and Avpr1b +⁄+ (**b**) males. **c**, **d** Representative photomicrographs of the anterior hypothalamic area (AHA) in Avpr1b −⁄− (**c**) and Avpr1b +⁄+ (**d**) males. The *arrows* indicate examples of EGR-1 immunopositive neurons at ×100 magnification and the *scale bar* represents 100 µm

In females, there were no statistically significant genotypic differences in EGR-1-immunoreactivity/mm^2^ in any of the brain regions examined (see Fig. 1a). However, in males there was a statistically significant genotypic difference in the number of EGR-1-ir cells/mm^2^ in the BNSTV (F_(1,20)_ = 6.89, p = 0.016) and the AHA (F_(1,18)_ = 8.46, p = 0.009). Specifically, within these two brain regions Avpr1b −/− males were found to have fewer EGR-1-ir cells/mm^2^ than wild type controls (see Figs. 1b, 2).

## Discussion

In this study we hypothesized that there would be genotypic differences in IEG activation in male and female Avpr1b −/− mice compared to wild type mice following a single encounter with an intruder. No genotypic differences in c-FOS-immunoreactivity were observed in females or males in any of the brain areas quantified, which is consistent with an earlier study by Wersinger et al. [17]. However, in males there was a significant genotypic difference in the expression of EGR-1 within the BNSTV and the AHA, both of which have been implicated in the neural circuitry regulating female and male aggression in rodents [as reviewed in 37]. Previous studies have also observed increases in c-FOS-immunoreactivity in these brain areas after intermale [39, 40] and maternal aggression [35]. Due in part to the low levels of aggressive behaviors displayed by Avpr1b −/− mice we examined intruder-evoked, not aggression-evoked, IEG activation. Though, previous work suggests that exposure to the context alone, in the absence of a physical encounter, is sufficient to result in neural activation in brain areas that are known to be important to aggressive behaviors [17, 34].

While we did not observe any IEG activation differences in female Avpr1b −/− mice compared to controls, this finding underscores the importance of the inclusion of females in this type of research as it suggests that the processing of an intruder social cue in female Avpr1b −/− mice differs from that of male Avpr1b −/− mice. Though, it is also important to consider that as we were only examining neuronal activation at a specific point in the postpartum period, it may be that a latter timepoint, or a different IEG would have yielded different results. Nonetheless, the sex difference is intriguing and future work should explore this, especially given that there is evidence that female Avpr1b −/− mice have decreased maternal aggression compared to controls [15].

With regards to the brain areas that have decreased EGR-1 immunoreactivity in male Avpr1b −/− mice, the BNST and the AHA are both a part of the social behavior neural network (SBNN) [4, 5, 41–43]. The MeA receives inputs from the olfactory bulbs and relays information to the MPOA, the lateral septum (LS), the AHA, and the BNST [40, 42]. Since Avpr1b expression is restricted to the CA2 region and not expressed in either the BNST or AHA, where and how hippocampal Avpr1b fits into this network is unknown. Avpr1b expression in the CA2 region [22] is thought to aid in the formation and or recall of memories related to social encounters; in particular those that are accessory olfactory system-based [1, 9, 11, 15]. Thus, the expression of the Avpr1b in the CA2 region is thought to be important for the determination of social context. Even though there was no genotypic difference in either the expression of c-FOS or EGR-1 within the CA2 region, this could indicate that the “social context” information is being relayed and integrated within some of the brain areas mentioned above to ultimately help determine an animal’s behavioral response. The lower levels of EGR-1 expression within the BNSTV and AHA of Avpr1b −/− males is likely due to an impairment in the transmission of this information. It is reasonable to hypothesize that genetic disruption of the Avpr1b alters the output from the CA2 region. Since the CA2 region projects to several brain areas known to be important in the SBNN, one possibility is that the CA2 projection to the LS [44], which lies up stream of the BNSTV and AHA, has been affected. This in turn could alter neuronal activity in the AHA as well as the BNSTV. It is also of note that downstream of the BNSTV is the ventral tegmental area, which is important to an animal’s motivational state [45], and perturbation of this signaling pathway would alter an animal’s behavioral output. This hypothesized interaction of the CA2 region with other brain regions within the SBNN is consistent with previous research demonstrating that lesions to the LS, the BNST or the AHA reduce intermale aggression [46]; there is also a role of Avp in the modulation of aggressive behavior in these brain regions, but those effects tend to be brain region- and Avpr1a-dependent [47–49].

It is also important to consider the possibility that other brain areas are important to the genotypic differences in aggressive behaviors that are observed in Avpr1b −/− mice compared to controls. Perhaps investigation of a different IEG would have revealed genotype-specific activation of additional brain areas or helped to identify putative neural substrates in females. Other phospho-proteins that may be indicators of neural activation include extracellular signal-regulated kinase (ERK) and phosphorylated cyclic AMP response element binding protein (pCREB); both of which are altered following an aggressive encounter [29, 50]. While these possibilities do not diminish the current findings, they are worthy of consideration in future studies.

By examining IEG activation in Avpr1b −/− and +/+ females and males following exposure to an intruder, we sought to identify what brain areas were “turned on” differentially between the genotypes, but it is important to consider the possibility that activation of the Avpr1b “turns off”, i.e. has inhibitory effects on the activity of specific brain regions. We chose to examine EGR-1, in addition to c-FOS, because EGR-1 expression has been found to differ from that of c-FOS following an aggressive encounter [30]. Based on the restricted expression of the Avpr1b to the CA2 region of the hippocampus we were hopeful that specific brain regions downstream of CA2 would be identified. It is important to note that the CA2 region is structurally and functionally distinct from other areas of the hippocampus [44, 51, 52], serving to link the CA1 and CA3 regions [53]. For example, it does not receive rich mossy fiber inputs from the dentate gyrus and lacks some of the morphology often observed in mossy fiber synapses [54, 55]. The CA2 region also exclusively expresses a variety of neurochemicals and receptors, such as fibroblast growth factor 2 [56], neurotrophin-3 [57], and the Avpr1b [22]. It is also the only part of the hippocampus to receive input from the posterior hypothalamus [58–60] and the perforant pathway; which connects the entorhinal cortex to the hippocampal formation [61]. Recent work has identified a novel Avpergic projection from the PVN to the CA2 region as well as one from CA2 pyramidal neurons to the supramammillary nuclei [44].

## Conclusions

Based on the results of this study it will be important to consider how the CA2 region may be a part of the neural circuitry that regulates social behaviors. As we are just now starting to understand the role of Avpr1b in the CA2 region future work will need to continue to focus on the functional connections between these numerous brain regions, their connection to and within the SBNN, and the phenotypes of the neurons that are activated following a social encounter. We are hopeful that these findings will help shed light on part of this connectivity and improve our understanding of a neural circuit that is likely to be important for displays of social behaviors in many species.

## Additional file

**Additional file 1.** Witchey et al. Data.xlsx contains the raw data for all of the experiments described in this manuscript.

## Abbreviations

AHA: anterior hypothalamic area
Avp: vasopressin
Avpr1a: vasopressin 1a receptor
Avpr1b: vasopressin 1b receptor
BNSTD: dorsal bed nucleus of the stria terminalis
BNSTV: ventral bed nucleus of the stria terminalis
EGR-1: early grown response factor 1
ERK: extracellular signal-regulated kinase
H_2_O_2_: hydrogen peroxide
IEG: immediate early gene
ir: immunoreactive
LS: lateral septum
LSD: dorsal lateral septum
LSV: ventral lateral septum
MeA: medial amygdala
PBS: phosphate buffered saline
pCREB: cyclic AMP response element binding protein
PND: postnatal day
PVN: paraventricular nucleus
SBNN: social behavior neural network
VTA: ventral tegmental area
−/−: knockout
